# Polymorphism studies on microRNA targetome of thalassemia

**DOI:** 10.6026/97320630014252

**Published:** 2018-05-31

**Authors:** Hamid Galehdari, Seyedeh Zohreh Azarshin, Mehdi Bijanzadeh, Mohammad Shafiei

**Affiliations:** 1 Thalassemia & Hemoglobinopathy Research center, research institute of Health, Ahvaz Jundishapur University of Medical Sciences, Ahvaz, Iran; 2 Department of Genetics, Faculty of Sciences, Shahid Chamran University of Ahvaz, Ahvaz, Iran

**Keywords:** MicroRNA, polymorphism, thalassemia

## Abstract

Thalassemia is one of the most prevalent hemoglobin disorders. It is caused by the decreased or absent synthesis of one globin chain that leads to moderate to severe hemolytic anemia in clinical complications. Some genetic factors cause these phenotypic variations by the alteration of gene expression. MicroRNAs (miRNAs) are post-transcriptional regulators in gene expression. Therefore, variations in 3'-untranslated region (3'-UTR) of target genes may affect gene expression. It is of interest to evaluate the impact of noncoding SNPs in thalassemia related genes on miRNA: mRNA interactions in the severity of thalassemia. Polymorphisms that alter miRNA: mRNA interactions were predicted using PolymiRTS and Mirsnpscore tools. Then, the effect of predicted target SNPs on thermodynamic stability, local RNA structure and regulatory elements was investigated using RNAhybrid, RNAsnp and RegulomeDB, respectively. The molecular functions and the Biological process of candidate genes were extracted and interaction network was created. Forty-six SNPs were predicted to affect 188 miRNA interactions. These results suggest that 3'-UTR SNP may affect gene expression and cause phenotypic variation in thalassemia patients.

## Background

Thalassemia is one of the most frequent inherited blood disorders with an autosomal recessive inheritance in the world. β- thalassemia is the result of decreased or complete absence of β- globin chain expression, leading to the imbalances of α/β rate. Excess alpha-chains precipitate in the erythroid cells and result in oxidative damage to the cell membrane, ineffective erythropoiesis and anemia [1]. Thalassemia intermedia are characterized by a wide clinical spectrum from the asymptomatic carrier state to more severe transfusion-dependent thalassemia major. Prediction of clinical severity in β-thalassemia intermedia patients is difficult due to some genetic factors that have been identified as modifying factors [2]. These factors cause phenotypic diversity, such as severity of anemia, bone marrow hyperplasia, need to blood transfusion and splenectomy requirements [3].

The primary modifying factors are the mild or silent mutations in beta-globin locus. These mutations lead to a smaller imbalance α/β globin ratio [4]. The second modifiers are co-inheritance α- thalassemia or genetic variations that can reduce free alphaglobin chains [4]. The product of some genes may reduce cytotoxic effects of free-alpha globin chains [5]. The third modifiers are variations that occur outside the globin gene cluster. These variations can affect bone, iron and bilirubin metabolism [3]. The free-alpha globin chains and iron overload can produce elevated levels of reactive oxygen species (ROS), resulting in oxidative stress in β-thalassemia. Oxidative stress leads to major complications but antioxidant enzymes protect cells against oxidative damage, such as catalase and glutathione S-transferases [6, 7]. Moreover, osteoporosis is one of the clinical symptoms of thalassemia that is responsible for morbidity in thalassemia major (TM) patients. The COLIA1 gene plays a major role in osteoporosis and has associated with low BMD in TM patients [8].

The gene expression can be controlled by multiple factors, such as transcription factors, activators, enhancers, repressors and translational factors. Among these factors, microRNAs (miRNAs) are the most important regulatory molecules that have a prominent role in post-transcriptional gene regulation. The dysregulation of miRNA may contribute to the pathogenesis of many human diseases, especially hematological disorders [9]. MiRNAs participate in controlling erythropoiesis and globin gene expression. Recent studies have reported that deregulation of miRNAs expression occurs in β-thalassemia disease. For example, miRNA-451 is up regulated in thalassemic erythroid progenitors. This up-regulation was correlated with disease severity in thalassemia [10]. MiR-486-3p inhibits expression of BCL11A by post-transcriptional mechanism and causes increased synthesis of HbF in adults [11]. MiRNAs are a class of small (-22 nucleotides long), non-coding RNAs. Studies have estimated that 60% of human protein-coding genes are regulated by miRNAs. Mature single strand miRNA is incorporated into the RNA-induced silencing complex (RISC) and recognizes 3'-untranslated regions (3'-UTRs) of target mRNAs by the Watson-Crick base pairing between seed region of miRNA and target site, leading to repression of mRNA translation or mRNA cleavage [12].

Genetic variations, such as single nucleotide polymorphisms (SNPs) in miRNA binding site of target mRNA may alter thermodynamics energy of RNA duplex and secondary structure of mRNA result in disrupting target site or creating new target site. Therefore, they can affect the regulation of target gene expression [9]. Polymorphisms in miRNA target site may have an association with susceptibility to many human diseases. For example, the rs1044129 within 3'-UTR of the RYR3 gene disrupts a binding site of mir-367 and has been associated with breast cancer risk [13]. The 3'-UTR SNP rs6573 in the PAP1A gene affects the binding site of mir-196a and leads to high expression of PAP1A gene [14]. However, phenotype prediction from genotype is very important for prenatal diagnosis and genetic counseling. To date, Correlation between genotype and phenotype is still unwell understood in TI disorder [15]. MiRNAs and variations in their binding site can moderate the clinical manifestation of diseases. Although, the effect of miRNA-target polymorphism has not been evaluated by both experimental and computational methods in TI disorder till now. The hypothesis of this study, SNPs that located in 3'-UTR of the targeted genes may affect miRNA: mRNA interaction, resulting in alteration of target gene expression and the subsequent clinical symptom of TI disorder. In the present study, SNPs that may alter the strength of miRNA: mRNA duplexes were predicted by in silico procedure. Then, the effective parameters on miRNA: mRNA interactions were evaluated for each SNP. Moreover, the functional role of candidate genes was identified.

## Methodology

**Figure 1:**
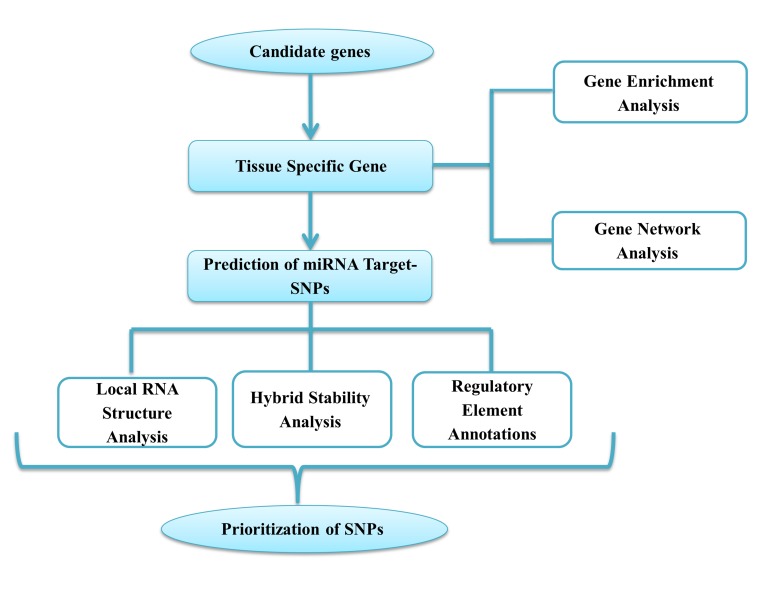
The workflow of bioinformatics procedure in this study

In this study, the genes related to the severity of thalassemia intermedia (TI) were extracted from known literatures [16, 17, 18, 19, 20]and the mRNA expression profile and molecular functions of these genes were obtained. Then, miRNA target site SNPs were predicted by in silico analysis. Furthermore, effective parameters in miRNA-target site-SNP (miR-TS-SNP) were analyzed. The workflow of this research was described in Figure 1.

### Extraction of gene expression profile

The expression profile of candidate genes was retrieved from TiGER (Tissue-specific Gene Expression and Regulation) (http://bioinfo.wilmer.jhu.edu/tiger/) to understand the pattern of gene expression and gene regulation in different tissues [21].

### MiroRNA target prediction

Polymorphism in miRNA target regions can result in the alteration in the miRNA: mRNA interaction. Therefore, the functional SNPs on miRNA target sites in the 3'-UTR of each gene were predicted by using PolymiRTS Database v3.0 (http://compbio.uthsc.edu/miRSN/) [22] and Mirsnpscore (http://www.bigr.medisin.ntnu.no/mirsnpscore/) [23].

### Effect of miRNA-target SNPs on miRNA: mRNA hybrid stability

The effect of target SNPs on the thermodynamic stability of miRNA: mRNA hybrid was defined with the ΔMFEhybrid score using the RNAhybrid web server (http://bibiserv.techfak.unibielefeld. de/rnahybrid/) [24]. For each interaction, a minimum free energy (MFE) of wild-type (WT) mRNA: miRNA hybrid and alternative (ALT) target mRNA:miRNA hybrid were measured, then the ΔMFEhybrid was computed as MFEhybrid(alt) - MFEhybrid(WT).

### Effect of miRNA-target SNPs on the mRNA secondary structures

The impact of miRNA-target SNPs on the local RNA secondary structure was predicted using the RNAsnp web tool (http://rth.dk/resources/rnasnp/) [25]. The structural difference between WT and variant alleles was calculated by using Euclidean distance or Pearson correlation measure for all sequence intervals. Then, the interval with maximum base pairing distance and the corresponding p-value were defined as outputs. Finally, SNPs with dmax p-value ≤ 0.2 were determined as structure disruptive variants.

### Functional annotation of the miRNA-target SNPs

Functional annotations were analyzed using HaploReg v4.1 (http://www.broadinstitute.org/mammals/haploreg/haploreg. php/) [26] and RegulomeDB (http://regulomedb.org) [27].

### Functional enrichment analysis

In other to identify the function of candidate genes and affected pathways by SNPs, the biological features were extracted from WebGestalt online tool kit (http://www.webgestalt.org/option.php/) [28] as follows; Gene ontology, gene-phenotype association and gene-disease association. Finally, the gene connection network was imaged using GeneMANIA plugin web app (http://www.genemania.org/plugin/) [29].

## Results

Most genes are expressed in specific tissues. Therefore, in order to make sure that the candidate genes are expressed in the affected tissues in thalassemia, the expression profile of each gene was obtained from TIGER. All of 26 genes were expressed in at least one of the affected tissues such as blood, bone, bone marrow, heart, liver and spleen (Table 1).

**Figure 2:**
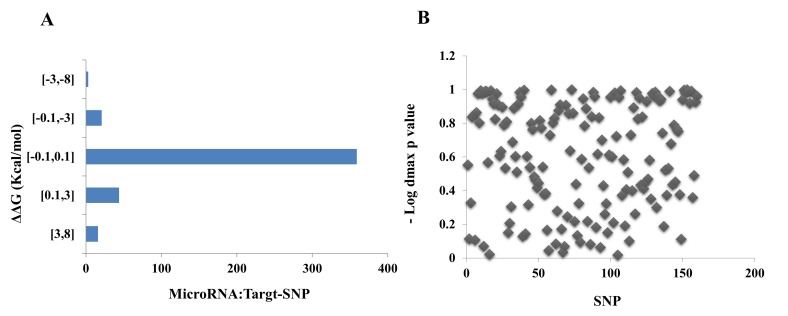
(A) The plot of ΔMFEhybrid for miRNA:mRNA (target SNPs) interactions. (B) The distribution plot of dmax p-value for target- SNPs by RNAsnp web tool. Each SNP with dmax P-value < 0.2 is significant.

Both PolymiRTS Database v3.0 and Mirsnpscore were used for predicting miRNA:mRNA target SNPs. The retrieved list of miRNA-target SNPs was screened to select modulating SNP. In this paper, target-SNP that may reduce the severity of clinical symptoms called modulating SNP. Therefore, SNPs were selected that disrupt miRNA binding sites in antioxidant enzyme genes (CAT, GSTs) and globin synthesis genes (HBB, HBG1 and HBG2). These SNPs may affect gene expression levels, leading to the elevated enzyme activity and the reduced imbalance α/β rate, respectively. The selected SNPs for AHSP and HRI genes also disrupt target sites that may result in low levels of alpha-globin, and prevent against the accumulation of alpha globin chains. In contrast, the target-SNPs were selected that create miRNA binding sites in BCL11A, HBA1 and HBA2 genes. These SNPs can lead to the elevated levels of HbF production and inhibit alphachain synthesis. Finally, 161 modulating SNPs for 415 miRNA were predicted that may affect 438 miRNA: mRNA interactions in 3'-UTR of 20 genes (supplementary file). Then, functional analysis was evaluated for these miRNA-target SNPs. SNPs in 3'- UTR region of the gene may create or disrupt the miRNAbinding site. Accordingly, RNAhybrid was used for studying the effect of target SNP on miRNA: mRNA hybrid stability. The negative value of ΔMFE demonstrates increase hybrid stability but the positive value of ΔMFE indicates the reduction of the hybrid stability. The stability of miRNA-target SNP duplex in 60 out of 438 interactions was reduced and in 25 interactions was increased and 353 miRNA-target SNP interactions were represented no change in the thermodynamic energy by target- SNPs (Figure 2A). RNAsnp was used to predict the effect of target-SNPs on the RNA structural change. Twenty-four target- SNPs out of 161 SNPs have the significant effect on the RNA secondary structure with dmax p-value ≤ 0.2 (Figure 2B, yellow cells in supplementary file).

SNPs in 3'-UTR of genes may affect the binding site of transcription factors. The result from HaploReg was revealed that the motif alteration was occurred in 146 out of 161 SNPs (supplementary file). Moreover, the further analysis was performed using RegulomeDB. Twenty-six SNPs had RegulomeDB score ≤ 3. Only rs11807 and rs4560 had score 1, likely to alter TF binding and have been associated with gene expression. However, other 24 SNPs had score 2 and 3 with less evidence for the effect on TF binding site (Table 2). Ninety SNPs had score ≥ 4 with the least binding evidence.

**Figure 3:**
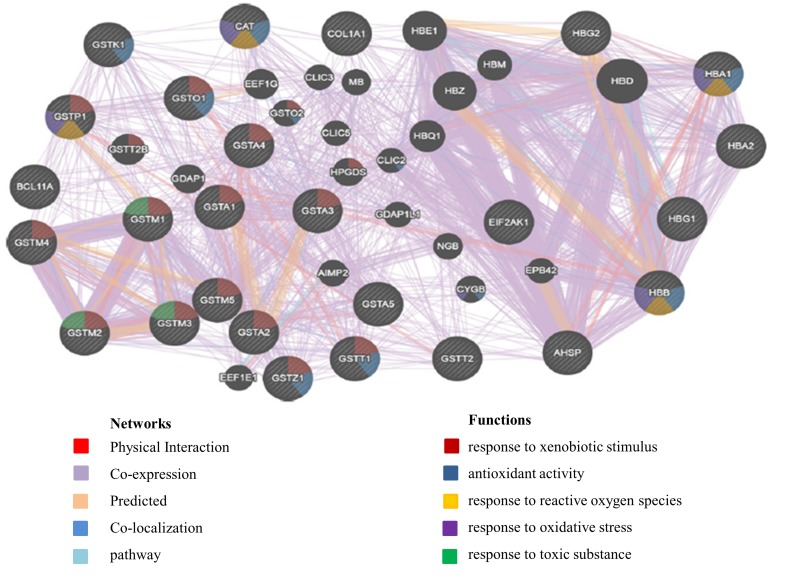
Network of gene-gene interaction derived from GeneMANIA. Circles with diagonal line represent the queried genes and circles without diagonal line represent mediated genes for interactions. These candidate genes represent co-expression, co-localization, predicted and physical interaction networks with themselves. some important functions of genes are shown with the color triangles in the circle: red triangle response to xenobiotic stimulus, blue triangle antioxidant activity, yellow triangle response to reactive oxygen species, violet triangle response to oxidative stress and green triangle response to toxic substance.

The molecular functions and biological process of candidate genes were identified using WebGestalt (Table 3). The functions, such as antioxidant activity, oxidoreductase activity, peroxidase activity and glutathione transferase activity are involved in detoxification ROS and xenobiotic compounds. Therefore, they can modulate some symptoms of thalassemia. One of the major causes of hemoglobinopathy is disruption of functions, like oxygen binding, heme binding and oxygen transporter activity. The analysis of gene-phenotype association demonstrated an abnormality in the heme biosynthesis and erythrocytes, splenomegaly and anemia. Therefore, expression of the candidate genes in the affected tissues can indicate the major role of these genes in the modulation of thalassemia severity. In addition, the gene network was constructed using the GeneMANIA server, under the biological process weighting. The result of network analysis indicated several networks between the set of candidate genes including, physical interactions, co-localization, coexpression and pathway. It confirms that variations in 3'-UTR region of each gene can affect these networks and phenotype severity related to thalassemia (Figure 3).

### Prioritization of the miRNA-target-SNPs

The candidate target-SNPs were prioritized which influence on the reduced severity of thalassemia symptoms. Accordingly, the target-SNPs were selected that caused more miRNA-mRNA interactions with Δ ≤ 0 and SNPs also altered binding motifs and TFs, and have likely effect on the gene expression. Finally, 46 SNPs were predicted as a potential reducer of thalassemia symptoms (Green cells in supplementary file).

## Discussion

Thalassemia is the hemolytic disorder with phenotypic heterogeneity and causes a wide range of symptoms [1]. Moreover, the understanding of the phenotype-genotype correlation is difficult due to modifying factors in thalassemia [2]. Several studies have been demonstrated that the occurring variation in some genes, such as HBA1, HBA2, HBB, HBG1, HBG2, BCL11A, COL1A1, CAT and GST genes can moderate the severity of thalassemia [16, 17, 18, 19, 20]. Recently, several studies have evaluated the effect of 3'-UTR SNPs on gene expression. These studies indicated an important role of miRNA-target-SNP on disease susceptibility [9, 30]. Furthermore, the computational approaches are useful for the prioritization of functional miRNA target-SNPs in biomedical research. The results of bioinformatics analysis predict probabilistic and potential candidate SNPs for further experimental approaches. Accordingly, this paper was evaluated by the hypothesis that 3'-UTR SNPs in thalassemia relevant genes may affect miRNA regulation and create phenotype diversity. Therefore, the bioinformatics approaches were used to identify functional 3'-UTR SNPs which reduce clinical severity of thalassemia.

First, the gene expression profile indicated all candidate genes have the expression in affected tissue in thalassemia such as, blood, spleen, heart and liver that it confirms variations in candidate genes may influence on the observed phenotype severity. Besides, these genes have an important role in molecular functions, such as antioxidant activity, oxidoreductase activity, peroxidase activity and glutathione transferase activity which are responsible for the protection against free radicals of oxygen, iron overload and reduce sensitivity to oxidative stress. The analysis of gene-phenotype association also indicates an abnormality in the heme biosynthesis and erythrocytes, splenomegaly and anemia by the disrupted candidate genes. List of miRNA target- SNPs was extracted using PolymiRTS and Mirsnpscore tools for each gene. Then, 161 modulating SNPs were selected from this list. In the next step, further analysis was performed: miRNAmRNA hybrid stability, annotation of TF and regulatory element. Twenty-six SNPs have RegulomeDB score ≤ 3 with likely effect on transcription factor binding. Only two SNPs rs11708 and rs4560 were found with the RegulomeDB score 1d and 1f, respectively and associated with gene expression. For example, the rs11807 in the GSTM5 gene was shown association with diagnosis age of Parkinson disease [31]. Another study indicated that SNP rs4560 could regulate gene expression in the lymphoblastoid cell [32]. However, the effect of variations on the local RNA secondary structure was investigated. Finally, out of 161 modulating SNPs, 55 SNPs of them were prioritized as high functional SNPs. Nine SNPs (rs4021970, rs12937105, rs75713851, rs4560, rs1050032, rs405729, rs1055259, rs145451237 and rs191030500) out of 55 functional SNPs were found with RNAsnp p-value ≤ 0.2 which can lead to disruption of RNA secondary structure and create a change in miRNA binding. Hence, there is a limitation for the understanding impact of the altered mRNA structure on how expression level of gene alters in this study. For this purpose, need to further experimental studies for predicting an apparent phenotype by these variations. For example, a recent study has shown that variation in pri-mir-30c-1 sequence can lead to alteration of miRNA secondary structure. This structural change promotes binding affinity of SR protein and increases expression of the mature miR-30c in breast cancer [33]. Another study had predicted rs75713851, which may have an effect on miRNA binding site and function of lung cancer-related genes [34]. Therefore, the disrupted structural SNPs were ignored from the prioritized functional SNPs. These results lead to the prioritization of 46 3'-UTR SNPs as modifying factors and 188 miRNA: mRNA interactions, which cause phenotype severity. Moreover, the findings are helpful to the understanding of the underlying mechanisms that miRNA-target SNPs regulate miRNA and alter gene expression. Moreover, these results contribute to genetic counseling for prenatal diagnosis and effective treatment of patients with thalassemia. However, further experimental validations and association studies should be performed to confirm the importance of these results.

## Conclusion

We report the predicted functional miRNA-target-SNPs. Results suggest that variations in miRNAtarget sites in thalassemia related genes might moderate phenotype severity of thalassemia. These non-coding SNPs can act as modifying factors to reduce α/β imbalance rate and cellular oxidative stress. Moreover, these modifiers contribute to the genetic counseling but more experimental studies need to indicate validation of these results.

## Supplementary data

Supplementary data

## Figures and Tables

**Table 1:** The candidate gene expression data in thalassemia uisng Expressed Sequence Tag (EST) Profile from Tiger

Gene	Tissue
HBA1	Spleen, Thymus, liver, Blood
HBA2	Spleen, Thymus, liver, Blood, Bone_Marrow,
HBB	Blood, Bone_Marrow, Muscle, Spleen
HBG1	Spleen, Thymus, Liver
HBG2	Spleen, Thymus, Liver
BCL11A	Lymph_Node, Thymus, Blood, Bone_marrow
EIF2AK1	Bladder, Blood, Spleen, Liver, Kidney, Bone, Bone_marrow, Testis
AHSP	Blood, Spleen, Bone_marrow, Heart, Liver
COL1A1	Bone, Bone_marrow, bladder, Heart, Spleen
CAT	Blood, Bone_marrow, Liver, Spleen
GSTA1	Liver, Testis
GSTA2	Liver, Testis, Kidney, Lung
GSTA3	Placenta, Liver, Testis
GSTA4	Heart, Kidney, PNS, Testis, Brain, Bone, Bone_marrow
GSTA5	Liver, Kidney, Testis
GSTM1	Spleen, Liver, Heart, Testis, Bladder, Bone
GSTM2	PNS, Ovary, Brain, Blood, Bone, Heart, Liver
GSTP1	Blood, bone, heart, spleen
GSTZ1	Bone, liver, heart, kidney
GSTM3	Blood, Testis, Liver, Bladder, Heart
GSTM4	Spleen. Stomach, Heart, Kidney, Liver, Blood, Bladder, Bone
GSTM5	Thymus, Heart, Brain
GSTT1	Liver, heart, Bladder, blood, Kidney, Bone, Thymus
GSTT2	Thymus, Heart, Liver
GSTO1	Spleen, Heart, Liver, Kidney, Bone, Skin
GSTK1	Blood, Bone, Bone_marrow, Heart, Liver, Spleen, Testis, Thymus

**Table 2:** List of miRNA target-SNPs in the binding of transcription factor

Gene	SNP ID	RegulomeDB score*
HBA1	rs3180978	2b
HBA2	rs4021968	2b
	rs4021969	2b
	rs3209669	2b
	rs4021965	2b
	rs3209698	2b
	rs4021970	3a
COL1A1	rs1061947	2a
	rs12937105	2b
	rs12944834	2b
	rs75713851	2b
	rs75168103	2b
	rs11556514	2b
	rs201085309	2b
	rs202170631	2b
	rs19806909	2b
	rs34162544	3a
HBG2	rs200786947	3a
HRI	rs3801032	2c
	rs4560	1f
GSTM5	rs11807	1d
	rs112412754	2b
	rs116803889	2b
	rs191030500	3a
GSTP1	rs5031031	3a
	rs201925035	3a
*It represents confidence for a variation to be resided in a functional location. This score was evaluated by supporting data as follow: 1d: eQTL + TF ((transcription factor)) binding + any motif + DNase peak; 1f: eQTL + TF binding / DNase peak; 2b: TF binding + any motif + DNase Footprint + DNase peak; 2c: TF binding + matched TF motif + DNase peak; 3a: TF binding + any motif + DNase peak.		

**Table 3:** Functional Enrichment annotation od candidate genes

Gene	SNP ID	RegulomeDB score*
HBA1	rs3180978	2b
HBA2	rs4021968	2b
	rs4021969	2b
	rs3209669	2b
	rs4021965	2b
	rs3209698	2b
	rs4021970	3a
COL1A1	rs1061947	2a
	rs12937105	2b
	rs12944834	2b
	rs75713851	2b
	rs75168103	2b
	rs11556514	2b
	rs201085309	2b
	rs202170631	2b
	rs19806909	2b
	rs34162544	3a
HBG2	rs200786947	3a
HRI	rs3801032	2c
	rs4560	1f
GSTM5	rs11807	1d
	rs112412754	2b
	rs116803889	2b
	rs191030500	3a
GSTP1	rs5031031	3a
	rs201925035	3a
aGene ontology; bRate of enrichment was retrieved from the WebGestalt online tool kit database, based on analyzing the ranked gene list and interactions among genes.
