# Supplementary material for: Polymorphism studies on microRNA targetome of thalassemia
Source: Bioinformation. 2018 May 31;14(5):252–8. doi: 10.6026/97320630014252 (PMC6077818; doi:10.6026/97320630014252)
Supplement: Supplementary data [file 97320630014252S1.pdf]

Supplementary file 1: List of target-SNP-miRNA interactions

Variant	Gene	Chr[1]	Position	Mir-RNA	Class[2]	MFE_{WT}	MFE_{MT}	ΔMFE[3]	Score of RNAsnp	RegulomeDB score	Motifs changed
rs3180978	*HBA1*	16	177450	hsa-miR-4271	C	-46.1	-47.5	-1.4	0.5531	2b	BCL, CHD2, HNF4, MAZR, MZF1, NF-kappaB, ZNF219, WT1, TFII-I,STAT,Rad21, Pbx3, PLAG1
				hsa-miR-4725-3p	C	-25.0	-24.4	0.6			
				hsa-miR-625-5p	C	-20.8	-22.9	-2.1			
rs3209627	*HBA2*	16	173621	hsa-miR-3928-3p	C	-31.6	-29.2	2.4	0.1145	4	CTCF, NRSF, PU.1, RXRA, Rad21, SMC3
rs3209623	*HBA2*	16	173619	hsa-miR-3928-3p	C	-31.6	-29.6	2	0.3278	4	PU.1, p300
rs3209669	*HBA2*	16	173641	hsa-miR-4493	C	-50.5	-50.5	0	0.8378	2b	Ik-1_2, Zfx
rs4021971	*HBA2*	16	173647	hsa-miR-7160-3p	C	-30.9	-30.9	0	0.8479	4	EWSR1-FLI1, Pax-5, VDR_4
rs4021970	*HBA2*	16	173649	hsa-miR-3184-5p	C	-29.2	-29.2	0	0.107	3a	SP1, Pou2f2, Pax-5, Myb, MAZR, Irf, Ets
				hsa-miR-423-5p	C	-27.5	-27.5	0			
rs4021969	*HBA2*	16	173662	hsa-miR-3179	C	-16.8	-20.3	-3.5	0.8638	2b	VDR_2, SP1, Pax-5, NRSF, NRSF
				hsa-miR-4696	C	-38.6	-40.6	-2			
rs4021968	*HBA2*	16	173664	hsa-miR-4708-3p	C	-21.4	-21.4	0	0.976	2b	CTCF, EWSR1-FLI1,MAZ, NRSF, PU.1, Pou2f2, Rad21,STAT, p300
rs4021965	*HBA2*	16	173676	hsa-miR-4459	C	-74.8	-74.8	0	0.8028	2b	AP-1, CTCF, Ets, NERF1a, NRSF, PU.1, RXRA, Rad21, SMC3, ZBTB7A
rs3209698	*HBA2*	16	173677	hsa-miR-4270	C	-60.5	-63.4	-2.9	0.9938	2b	Rad21, NERF1a, ,ZBTB7A MAZ
				hsa-miR-4441	C	-56.7	-59.6	-2.9			
				hsa-miR-6753-5p	C	-28.3	-29.3	-1			
				hsa-miR-6754-5p	C	-38.1	-41.0	-2.9			

rs63750067	*HBA2*	16	173692	hsa-miR-4272	C	-24.9	-28.6	-3.7	0.9779	4	Hbp1,Egr-1
rs12937105	*COL1A1*	17	50184137	hsa-miR-4705	C	-51.7	-51.7	0	0.07	2b	Pou2f2, Ncx, Nkx6, Pax, Msx, Hox, Hlx1, En-1, Dlx, CDP, Arid3a Zfp105, Sox, Pou4f3, Pou3f4, Pou3f2_4
rs12944834	*COL1A1*	17	50184194	hsa-miR-4790-5p	C	-49.4	-49.4	0	0.9905	2b	TATA, TCF12, STAT, RXRA, PU.1, Irf, EWSR1-FLI1
				hsa-miR-6830-3p	C	-71.6	-71.6	0			
rs73987442	*COL1A1*	17	50184269	hsa-miR-1286	C	-73.7	-73.7	0	0.9897	4	ERalpha-a
				hsa-miR-3605-5p	C	-91	-91	0			
				hsa-miR-4722-5p	C	-77.3	-77.3	0			
rs34162544	*COL1A1*	17	50184311	hsa-miR-6880-5p	C	-35.8	-35.8	0	0.5685	3a	E2A_1, LBP- ,AP-4, Ascl2 1, Lmo2-complex_1
rs75713851	*COL1A1*	17	50184327	hsa-miR-151a-5p	C	-84.6	-84.6	0	0.0219	2b	HNF4, pz1
				hsa-miR-151b	C	-84.6	-84.6	0			
rs1062135	*COL1A1*	17	50184349	hsa-miR-6768-3p	C	-30.5	-30.5	0	0.9939	4	BDP1 ,RXRA, HNF4
rs1061947	*COL1A1*	17	50184758	hsa-miR-1224-3p	C	-37.6	-37.6	0	0.9442	2a	E2F, GR, ,AP-2, BCL P300, SP1
				hsa-miR-1260a	C	-79.6	-79.6	0			
				hsa-miR-1260b	C	-86.2	-86.2	0			
				hsa-miR-150-5p	C	-33.5	-33.5	0			
				hsa-miR-2116-3p	C	-30.1	-30.1	0			
				hsa-miR-4713-5p	C	-33.2	-33.2	0			
				hsa-miR-500b-3p	C	-27.9	-27.9	0			
				hsa-miR-532-3p	C	-37.5	-37.5	0			
				miR-1912	C	-61.8	-61.8	0			
				miR-188-3p	C	-29.4	-29.4	0			
				miR-3130-5p	C	-31.6	-31.6	0			

				miR-150	C	-95.1	-95.1	0			
rs199723627	*COL1A1*	17	50184819	hsa-miR-142-3p	C	-23.0	-25.1	-2.1	0.9168	4	IRC900814
				hsa-miR-3674	C	-54.4	-54.4	0			
				hsa-miR-3677-5p	C	-34.4	-34.4	0			
				hsa-miR-6073	C	-75.9	-75.9	0			
rs77720683	*COL1A1*	17	50184820	hsa-miR-328-3p	C	-36.2	-36.2	0	0.8252	4	IRC900814, Nanog
				hsa-miR-431-5p	C	-25.9	-25.9	0			
				hsa-miR-511-5p	C	-25.7	-25.7	0			
rs75168103	*COL1A1*	17	50185023	hsa-miR-1238-5p	C	-34.5	-34.5	0	0.977	2b	PLAG1, Nkx2
				hsa-miR-3659	C	-76.8	-76.8	0			
				hsa-miR-4658	C	-69.8	-69.8	0			
				hsa-miR-4758-5p	C	-37.4	-37.4	0			
				hsa-miR-574-5p	C	-38.8	-38.8	0			
				hsa-miR-6790-5p	C	-31.2	-31.2	0			
rs11556514	*COL1A1*	17	50185470	hsa-miR-1193	C	-70.4	-70.4	0	0.9115	2b	EWSR1-FLI1, Egr-1 ,Duxl
				hsa-miR-432-3p	C	-32.6	-32.6	0			
				hsa-miR-6871-5p	C	-34.3	-34.3	0			
rs201085309	*COL1A1*	17	50185481	hsa-miR-1321	C	-25.8	-25.8	0	0.6073	2b	TLX1, TCF12, ,TFII-I EWSR1-FLI1
				hsa-miR-1827	C	-57.4	-57.4	0			
				hsa-miR-3612	C	-92.5	-92.5	0			
				hsa-miR-4736	C	-57.6	-57.6	0			
				hsa-miR-4739	C	-110.9	-110.9	0			
				hsa-miR-4756-5p	C	-34.2	-34.2	0			
				hsa-miR-650	C	-111.7	-111.7	0			
				hsa-miR-657	C	-106.4	-106.4	0			
				hsa-miR-6808-5p	C	-37.0	-37.0	0			

				hsa-miR-6893-5p	C	-37.1	-37.1	0			
				hsa-miR-940	C	-114.5	-114.5	0			
rs199806909	*COL1A1*	17	50185482	hsa-miR-4300	C	-72.0	-72.0	0	0.6339	2b	TLX1, EWSR1- ,Zfp410 FLI1
				hsa-miR-4633-3p	C	-29.4	-29.4	0			
				hsa-miR-5591-5p	C	-27.4	-27.4	0			
				hsa-miR-6500-5p	C	-31.9	-31.9	0			
				hsa-miR-6726-5p	C	-36.1	-36.1	0			
				hsa-miR-920	C	-63.1	-63.1	0			
rs202170631	*COL1A1*	17	50185486	hsa-miR-4505	C	-81.5	-81.5	0	0.8966	2b	Pax-5, ,Zfp410, STAT WSR1-FLI1
				hsa-miR-4755-3p	C	-29.5	-29.5	0			
				hsa-miR-5787	C	-76.7	-76.7	0			
				hsa-miR-619-5p	C	-30.2	-30.2	0			
				hsa-miR-6506-5p	C	-26.1	-26.1	0			
rs1061237	*COL1A1*	17	50185414	miR-4260	C	-28.3	-28.3	0	0.7525	5	BTB7A, RREB- Zfp740,Z 1, Ik-1
				miR-1226*	C	-83.4	-83.4	0			
				miR-1304	C	-84.3	-84.3	0			
rs33985472	*HBB*	11	5225485	hsa-miR-4307	D	-35.9	-36.6	-0.7	0.79	5	Hox, Fox, ,Zfp105, PLZF DMRT2, Barhl1
rs34029390	*HBB*	11	5225502	hsa-miR-488-5p	D	-21.3	-17.9	3.4	0.5342	5	Hand1 ,Spdef, RP58
rs193922549	*HBB*	11	5225507	hsa-miR-7158-5p	D	-27.3	-24.4	2.9	0.8098	5	LRH1, ,TAL1, RP58 Hand1, GCNF
rs200399660	*HBB*	11	5225564	hsa-miR-3914	D	-21.3	-16.4	4.9	0.1514	5	SRF, DMRT2 ,TCF4, Sox
rs113910406	*HBG1*	11	5248325	hsa-miR-362-5p	D	-27.8	-27.2	0.6	0.2071	5	CTCF,P300
				hsa-miR-500b-5p	D	-19.7	-19.1	0.6			
				hsa-miR-501-5p	D	-21.7	-21.7	0			
rs200786947	*HBG2*	11	5253260	hsa-miR-20a-3p	D	-23.6	-17.0	6.6	0.305	3a	GATA ,Znf143, NRSF
				hsa-miR-3194-3p	D	-31.3	-26.9	4.4			
rs2058703	*BCL11A*	2	60452091	miR-300	C	-58.4	-58.4	0	0.69	no data	

				miR-381	C	-50.5	-50.5	0			
rs3198285	*BCL11A*	2	60457239	hsa-miR-6751-5p	C	-30.8	-32.6	-1.8	0.8892	6	AIRE,Gcm1,RXRA
				hsa-miR-6752-5p	C	-36.4	-36.4	0			
				hsa-miR-6803-5p	C	-39.2	-39.2	0			
				hsa-miR-6835-5p	C	-27.2	-27.2	0			
				hsa-miR-6842-5p	C	-27.1	-27.1	0			
				hsa-miR-7110-5p	C	-34.0	-34.0	0			
rs12986851	*BCL11A*	2	60457292	hsa-miR-4446-5p	C	-25.5	-25.5	0	0.6035	6	Ik-1, Ik-2, NF-AT
rs112294072	*BCL11A*	2	60457363	hsa-miR-4427	C	-47.8	-47.8	0	0.5111	6	Foxo, Foxp,HDAC2,HNF4, Mef2, PPAR, Pou5f1, TATA, Zfp, p300
				hsa-miR-452-3p	C	-23.2	-23.2	0			
				hsa-miR-4680-3p	C	-17.8	-17.8	0			
				hsa-miR-5187-3p	C	-23.6	-23.6	0			
rs187957411	*BCL11A*	2	60457372	hsa-miR-548c-3p	C	-16.5	-16.5	0	0.9157	6	Cdx, Evi-1, Fox, HDAC2, HMG-IY, HNF1, Irf, Mef2, Pou2f, TATA, Zfp, p300
rs192239475	*BCL11A*	2	60457373	hsa-miR-3163	C	-44.3	-44.3	0	0.9847	6	Cdx, Dbx1,Evi-1, Foxa, Foxd, Foxo, Foxp, HDAC2, HMG-IY, HNF1, Hoxa, Irf, Ncx, Pou5f1, TATA,Zfp, p300
rs73932586	*BCL11A*	2	60457486	hsa-miR-425-5p	C	-18.6	-18.6	0	0.9552	6	IRC900814
rs79537300	*BCL11A*	2	60457672	hsa-miR-4694-5p	C	-22.9	-22.9	0	0.1296	6	FAC, Foxp1, HDAC2, HMG-IY, Irf,Pax-4, Zfp, p300
rs78623367	*BCL11A*	2	60457952	hsa-miR-4753-3p	C	-21.9	-21.9	0	0.997	6	Foxp1, HDAC2, Irf, Pax-5, RXRA, STAT,p300
				hsa-miR-6809-3p	C	-23.4	-23.4	0			
rs113804646	*BCL11A*	2	60458459	hsa-miR-4282	C	-46.0	-46.0	0	0.1447	No Data	Ncx,Pou2f2,TATA

rs185027121	*BCL11A*	2	60458573	hsa-miR-148a-5p	C	-21.0	-21.0	0	0.6039	No Data	Arid5b,RXR::LXR
rs111694591	*BCL11A*	2	60459047	hsa-miR-128-3p	C	-19.8	-19.8	0	0.3165	6	HNF1,Pou2f2,Pou3f2
				hsa-miR-216a-3p	C	-22.5	-22.5	0			
				hsa-miR-27a-3p	C	-21.6	-21.6	0			
				hsa-miR-27b-3p	C	-21.3	-21.3	0			
				hsa-miR-3681-3p	C	-24.9	-24.9	0			
				hsa-miR-513a-5p	C	-25.4	-25.4	0			
rs1052520	*BCL11A*	2	60459153	hsa-miR-1237-3p	C	-25.6	-25.6	0	0.5393	No Data	SZF1, Spz1, Tgif1, p300
				hsa-miR-204-5p	C	-31.3	-27.7	3.6			
				hsa-miR-211-5p	C	-32.6	-29.0	3.6			
				hsa-miR-3182	C	-49.7	-49.7	0			
				hsa-miR-4743-3p	C	-23.9	-24.3	-0.4			
				hsa-miR-4755-5p	C	-28.4	-28.7	-0.3			
				hsa-miR-5006-3p	C	-25.4	-25.4	0			
				hsa-miR-5088-3p	C	-25.7	-26.0	-0.3			
				hsa-miR-623	C	-72.1	-72.1	0			
				hsa-miR-642b-5p	C	-26.7	-26.7	0			
				hsa-miR-6888-3p	C	-22.4	-21.0	1.4			
rs2287086	*BCL11A*	2	60459360	hsa-miR-153-3p	C	-19.3	-19.3	0	0.8010	6	Nanog,Pou2f2,TATA
				hsa-miR-3680-3p	C	-25.6	-25.6	0			
				hsa-miR-448	C	-73.3	-73.3	0			
				hsa-miR-6504-3p	C	-25.1	-25.1	0			
rs192344559	*BCL11A*	2	60459545	hsa-miR-3529-5p	C	-27.3	-27.3	0	0.7641	5	Foxj2,Smad,Sox
				hsa-miR-379-5p	C	-23.2	-23.2	0			
				hsa-miR-3927-3p	C	-24.0	-24.0	0			
				hsa-miR-6831-5p	C	-31.2	-31.2	0			
rs142636119	*BCL11A*	2	60459548	hsa-miR-1208	C	-53.8	-53.8	0	0.4833	5	Sox

				hsa-miR-4252	C	-51.7	-51.7	0			
				hsa-miR-449b-3p	C	-21.6	-21.6	0			
rs150974916	*BCL11A*	2	60459702	hsa-miR-1252-3p	C	-19.2	-19.2	0	0.4659	No Data	PLZF
				hsa-miR-3123	C	-47.0	-47.0	0			
				hsa-miR-4712-3p	C	-23.2	-23.2	0			
rs141136901	*BCL11A*	2	60459795	hsa-miR-32-3p	C	-20.2	-20.2	0	0.4180	5	Smad3
rs74493918	*BCL11A*	2	60459806	hsa-miR-3919	C	-59.9	-59.9	0	0.4450	5	
				hsa-miR-4756-3p	C	-26.2	-26.2	0			
				hsa-miR-6854-5p	C	-26.2	-26.2	0			
rs112094046	*BCL11A*	2	60459930	hsa-miR-5681b	C	-45.0	-45.0	0	0.8170	5	HDAC2
rs181979594	*BCL11A*	2	60459950	hsa-miR-130b-5p	C	-20.8	-20.8	0	0.7727	5	BCL, Pax-5, TATA, p300
				hsa-miR-6817-3p	C	-24.7	-24.7	0			
				hsa-miR-6873-3p	C	-36.5	-37.0	-0.5			
				hsa-miR-7110-3p	C	-22.5	-22.5	0			
rs185460868	*BCL11A*	2	60459984	hsa-miR-129-5p	C	-25.4	-25.4	0	0.5411	5	DBP, DMRT1, DMRT1, HDAC2, HNF4, Irx, TCF12, p300
				hsa-miR-153-5p	C	-25.0	-25.0	0			
				hsa-miR-6885-3p	C	-24.1	-24.1	0			
rs180821769	*BCL11A*	2	60460124	hsa-miR-4255	C	-52.0	-52.0	0	0.3796	4	CHOP::CEBPalpha,p300
				hsa-miR-4423-5p	C	-22.2	-22.2	0			
				hsa-miR-6501-5p	C	-29.4	-29.4	0			
				hsa-miR-744-3p	C	-21.4	-21.4	0			
rs3801032	*HRI*	7	6022897	miR-3925	D	-58.1	-58.1	0	0.3837	2c	GATA, TAL1,Pou5f1,HMGN3,HDAC2
				miR-1253	D	-71.2	-71.2	0			
				miR-3123	D	-52.8	-52.8	0			
rs3801030	*HRI*	7	6022598	miR-3168	D	-66.9	-66.9	0	0.1659	5	GATA
rs4560	*HRI*	7	6023652	miR-619	D	-89.8	-89.8	0	0.0447	1f	

				miR-221*	D	-21.0	-20.6	0.4			
				miR-490-3p	D	-24.2	-23.0	1.2			
rs10843	*AHSP*	16	31528709	miR-3916	D	-56.2	-56.1	0.1	0.7297	4	CCNT2, E2F, Egr-1, Irf, MAZR, SP1, TCF12, Zfp, ZNF
				miR-3125	D	-49.1	-49.1	0			
				miR-3150b	D	-68.0	-67.1	0.9			
				miR-423-5p	D	-27.3	-27.9	-0.6			
				miR-1914*	D	-55.0	-52.9	2.1			
				miR-3184	D	-37.9	-37.9	0			
rs148412737	*AHSP*	16	31528759	hsa-miR-4451	D	-39.9	-39.3	0.6	0.9985	4	COMP1, HNF4, Pbx3, ZBTB33
				hsa-miR-5586-3p	D	-21.8	-19.6	2.2			
rs148299275	*CAT*	11	34471486	hsa-miR-125b-1-3p	D	-25.0	-25.0	0	0.803	No Data	ATF3,Duxl,Pax-8
rs141451783	*CAT*	11	34471512	hsa-miR-4768-3p	D	-23.7	-21.7	2	0.8295	No Data	
				hsa-miR-585-5p	D	-23.2	-21.3	1.9			
rs11546300	*CAT*	11	34471593	hsa-miR-1179	D	-54.1	-54.1	0	0.0843	No Data	DMRT, Pou2f2, Sox
rs2266625	*CAT*	11	34471729	hsa-miR-590-3p	D	-16.6	-16.6	0	0.2795	No Data	
rs1050028	*CAT*	11	34471763	hsa-miR-190a-5p	D	-18.8	-18.8	0	0.8759	No Data	Foxa, Foxj2, Mef2, SIX5, Znf143
				hsa-miR-190b	D	-40.1	-40.1	0			
				hsa-miR-3689a-5p	D	-24.2	-24.2	0			
				hsa-miR-3689b-5p	D	-24.2	-24.2	0			
				hsa-miR-3689e	D	-56.5	-56.5	0			
				hsa-miR-3689f	D	-56.2	-56.2	0			
				hsa-miR-7854-3p	D	-19.7	-19.7	0			
				hsa-miR-6134	D	-72.1	-72.1	0			
rs2266629	*CAT*	11	34471898	hsa-miR-3182	D	-46.0	-46.0	0	0.9103	No Data	GATA
rs1050032	*CAT*	11	34471909	hsa-miR-7158-5p	D	-25.6	-28.0	-2.4	0.1717	No Data	
				hsa-miR-3907	D	-93.2	-93.2	0			

rs193228551	*CAT*	11	34471993	hsa-miR-512-3p	D	-23.9	-23.9	0	0.0346	6	CEBPG, Gfi1, Gfi1b, Hdx
				hsa-miR-148a-3p	D	-20.1	-18.1	2			
				hsa-miR-148b-3p	D	-19.4	-17.7	1.7			
				hsa-miR-152-3p	D	-21.9	-18.3	3.6			
				hsa-miR-5094	D	-42.0	-42.0	0			
rs80205455	*CAT*	11	34472012	hsa-miR-7641	D	-21.3	-21.3	0	0.0693	6	Dbx, Hmx, Hoxb, Hoxd, Ncx
rs111528251	*GSTA1*	6	52791417	hsa-miR-943	D	-72.5	-72.5	0	0.9081	6	BATF, Bcl6b, Foxp3, Hoxa, Nrf1,STAT
rs1052128	*GSTA1*	6	52791624	hsa-miR-665	D	-65.2	-65.2	0	0.246	6	AIRE, Bcl6b, ELF1, Pax-5, STAT,
	GSTA1	6	52791630	hsa-miR-522-3p	D	-18.5	-18.5	0	0.8589	6	Bcl6b, CEBPB, Myb, STAT
				hsa-miR-224-3p	D	-19.8	-19.8	0			
rs1052119	*GSTA1*	6	52791631	hsa-miR-224-3p	D	-19.8	-19.8	0	0.6372	6	Bcl6b, CEBPB, NF-AT, STAT
				hsa-miR-522-3p	D	-18.5	-18.5	0			
rs1052116	*GSTA1*	6	52791655	hsa-miR-4427	D	-40.6	-40.6	0	1	No Data	CEBPB, Evi, HEY1, NF-AT, STAT
				hsa-miR-4680-3p	D	-17.8	-17.8	0			
				hsa-miR-5093	D	-59.5	-59.5	0			
				hsa-miR-5187-3p	D	-18.5	-18.5	0			
rs189610458	*GSTA1*	6	52791662	hsa-miR-130a-5p	D	-19.1	-19.1	0	0.8609	No Data	HEY1
				hsa-miR-23a-3p	D	-19.0	-19.0	0			
				hsa-miR-23b-3p	D	-19.0	-19.0	0			
				hsa-miR-23c	D	-56.1	-56.1	0			
				hsa-miR-4452	D	-53.5	-53.5	0			
				hsa-miR-4680-3p	D	-17.8	-17.8	0			
rs117087749	*GSTA1*	6	52791671	hsa-miR-5100	D	-75.2	-73.5	1.7	0.2177	No Data	Mef2,Pou2f2
				hsa-miR-1288-5p	D	-24.6	-24.6	0			
rs699334	*GSTA1*	6	52791798	hsa-miR-5701	D	-15.3	-15.3	0	0.4399	6	DMRT, Dbx2, Hmx, Hoxd, Ncx, Nkx,Pax, Sox,

rs73740640	*GSTA1*	6	52791801	hsa-miR-744-3p	D	-15.1	-15.1	0	0.1335	6	CEBPB, DMRT4, Nkx, Sox,p300
				hsa-miR-5701	D	-15.3	-15.3	0			
rs1052027	*GSTA1*	6	52791819	hsa-miR-5687	D	-34.1	-31.0	3.1	0.3247	No Data	HP1-site-factor
rs699336	*GSTA1*	6	52791832	hsa-miR-5584-3p	D	-16.6	-16.1	0.5	0.0943	No Data	CHOP::CEBPalpha,GR
				hsa-miR-148a-5p	D	-22.0	-19.9	2.1			
rs1052009	*GSTA1*	6	52791840	hsa-miR-6878-3p	D	-20.1	-17.4	2.7	0.5879	No Data	FXR, Eralpha, GCNF.HMG-IY, PPAR,Pax-5,RORalpha
				hsa-miR-4757-5p	D	-31.2	-28.5	2.7			
				hsa-miR-6744-3p	D	-24.3	-21.6	2.7			
rs73740641	*GSTA1*	6	52791841	hsa-miR-6878-3p	D	-20.1	-14.7	5.4	0.9465	No Data	Pax-5,ZID
				hsa-miR-6744-3p	D	-24.3	-20.5	3.8			
				hsa-miR-4757-5p	D	-31.2	-24.0	7.2			
rs1052003	*GSTA1*	6	52791849	hsa-miR-5002-3p	D	-17.8	-15.5	2.3	0.785	No Data	Pax-5
rs41271283	*GSTA2*	6	52750159	hsa-miR-6830-3p	D	-22.5	-22.5	0	0.8884	No Data	HMG-IY
				hsa-miR-511-5p	D	-19.3	-19.3	0			
rs140525011	*GSTA2*	6	52750432	hsa-miR-4433b-3p	D	-27.9	-22.8	5.1	0.2181	No Data	Ets, Pax,Pou3f1, RXRA
rs114264491	*GSTA2*	6	52750439	hsa-miR-4795-3p	D	-19.1	-19.1	0	0.5366	6	BAF155, Dbx, En, Ets, Foxa, Foxd, Hlx, Hox, Lhx3, Ncx, Pax, Pou2f2, RXRA, Sox
				hsa-miR-126-5p	D	-18.0	-18.0	0			
rs1803683	*GSTA2*	6	52750521	hsa-miR-4684-3p	D	-23.4	-19.8	3.6	0.0816	6	CEBPA, DMRT, Hmx, Hoxd,Nkx, Pax, Pou3f, Sox,TATA,p300
				hsa-miR-744-3p	D	-15.4	-15.2	0.2			
rs199647015	*GSTA2*	6	52750547	hsa-miR-5011-3p	D	-19.6	-19.6	0	0.8394	No Data	

				hsa-miR-4666b	D	-42.1	-42.1	0			
rs2266632	*GSTA2*	6	52750555	hsa-miR-4677-5p	D	-19.3	-19.3	0	0.9841	No Data	Eralpha, FXR, GCNF, Pou2f2
				hsa-miR-2116-5p	D	-22.1	-22.1	0			
				hsa-miR-22-5p	D	-18.9	-18.9	0			
rs45451796	*GSTA3*	6	52896799	miR-6762-3p	D	-28.7	-28.7	0	0.959	No Data	Arid3a, Mef2, Mrg1::Hoxa9, YY1
				hsa-miR-889-5p	D	-18.0	-19.3	-1.3			
				hsa-miR-604	D	-48.6	-48.6	0			
				hsa-miR-647	D	-66.1	-68.9	-2.8			
rs405729	*GSTA4*	6	52977983	hsa-miR-8084	D	-39.7	-39.7	0	0.1828	No Data	Foxp1, Mef2
				miR-141	D	-21.7	-21.7	0			
				miR-200a	D	-21.6	-21.6	0			
				hsa-miR-200b-3p	D	-18.3	-18.3	0			
				hsa-miR-200c-3p	D	-20.2	-20.2	0			
				hsa-miR-429	D	-57.9	-57.9	0			
rs45547837	*GSTA4*	6	52977986	hsa-miR-8084	D	-39.7	-39.7	0	0.6174	6	CHOP::CEBPalpha,NF-I
				hsa-miR-200b-3p	D	-18.3	-18.3	0			
				hsa-miR-200c-3p	D	-20.2	-20.2	0			
				hsa-miR-429	D	-57.9	-57.9	0			
rs7496	*GSTA4*	6	52978041	hsa-miR-4305	D	-61.3	-61.3	0	0.834	No Data	Hmbox1,Nanog
				miR-28-3p	D	-21.3	-21.3	0			
rs185167676	*GSTA4*	6	52978125	hsa-miR-4468	D	-49.7	-49.7	0	0.0629	No Data	ELF1, TATA, TCF12, p300
rs151009303	*GSTA4*	6	52978324	hsa-miR-6088	D	-44.3	-44.3	0	0.701	No Data	Bcl6b,STAT
				hsa-miR-5197-3p	D	-21.8	-20.2	1.6			
				hsa-miR-4770	D	-45.6	-45.6	0			
				hsa-miR-143-3p	D	-22.4	-22.4	0			
rs72550711	*GSTA4*	6	52978361	hsa-miR-711	D	-60.2	-58.6	1.6	0.4307	No Data	

rs200050792	*GSTA4*	6	52978439	hsa-miR-3914	D	-18.5	-18.5	0	0.261	6	Bcl6b,GR
rs367836	*GSTA4*	6	52978333	miR-130b*	D	-64.6	-64.6	0	0.3237	No Data	
				miR-3675-3p	D	-23.4	-23.4	0			
rs17614751	*GSTA4*	6	52978051	miR-323b-5p	D	-20.9	-20.9	0	0.1514	No Data	Arid3a,BRCA1
rs4331985	*GSTA5*	6	52831777	hsa-miR-6509-5p	D	-19.8	-19.8	0	0.6127	No Data	Nkx2,Nkx3
				hsa-miR-331-5p	D	-23.3	-20.5	2.8			
rs201824944	*GSTA5*	6	52831792	hsa-miR-744-3p	D	-13.9	-12.4	1.5	0.9555	6	DMRT, Nkx, Sox
				hsa-miR-4289	D	-50.1	-50.1	0			
				hsa-miR-4684-3p	D	-22.8	-20.7	2.1			
rs185284510	*GSTA5*	6	52831829	hsa-miR-6878-3p	D	-24.0	-24.0	0	0.6015	No Data	
				hsa-miR-4757-5p	D	-21.9	-19.8	2.1			
				hsa-miR-6744-3p	D	-22.8	-21.3	1.5			
rs145996121	*GSTA5*	6	52831833	hsa-miR-6878-3p	D	-24.0	-23.7	0.3	0.2103	No Data	SEF-1,ZBTB7A
				hsa-miR-4757-5p	D	-21.9	-21.0	0.9			
				hsa-miR-6744-3p	D	-22.8	-22.3	0.5			
rs56328314	*GSTM3*	1	109733943	hsa-miR-4436b-3p	D	-27.6	-27.6	0	0.9809	5	EWSR1-FLI1,STAT
				hsa-miR-4632-5p	D	-30.9	-30.9	0			
				hsa-miR-4761-3p	D	-29.0	-29.0	0			
				hsa-miR-6735-5p	D	-33.7	-33.7	0			
				hsa-miR-6869-5p	D	-35.0	-35.0	0			
				hsa-miR-6879-5p	D	-33.0	-30.3	2.7			
				hsa-miR-7843-5p	D	-32.4	-32.4	0			
rs112564505	*GSTM3*	1	109734187	hsa-miR-1273d	D	-82.8	-82.8	0	0.7233	5	AP-1

				hsa-miR-548as-3p	D	-20.4	-20.4	0			
rs1055259	*GSTM3*	1	109734239	hsa-miR-3160-3p	D	-27.2	-27.2	0	0.0179	5	Mrg1::Hoxa9, NRSF, Pdx1,Pou5f1, Pou6f1, Sox
				hsa-miR-374a-5p	D	-18.4	-18.4	0			
				hsa-miR-374b-5p	D	-22.5	-22.5	0			
				hsa-miR-4487	D	-72.5	-72.5	0			
				hsa-miR-556-5p	D	-23.8	-23.8	0			
				hsa-miR-558	D	-75.0	-75.0	0			
rs56079534	*GSTM3*	1	109734369	hsa-miR-2682-5p	D	-31.1	-31.1	0	0.9541	No Data	KAP1
				hsa-miR-34b-5p	D	-30.1	-30.1	0			
				hsa-miR-449c-5p	D	-29.8	-29.8	0			
rs184868354	*GSTM3*	1	109734422	hsa-miR-1976	D	-58.4	-58.4	0	0.9943	No Data	EBF, ERalpha-a, GATA, Ik-2
				hsa-miR-4279	D	-71.6	-71.6	0			
				hsa-miR-5088-3p	D	-35.8	-35.1	0.7			
rs190061256	*GSTM3*	1	109734451	hsa-miR-7151-5p	D	-28.6	-28.6	0	0.3721	6	sl2,Zfp410
rs146748975	*GSTM3*	1	109734488	hsa-miR-3941	D	-68.0	-68.0	0	0.5841	6	Cdx2,Hoxb, Nrf-2, Rhox
				hsa-miR-466	D	-44.2	-44.2	0			
				hsa-miR-4672	D	-85.2	-85.2	0			
				hsa-miR-6768-5p	D	-26.3	-26.3	0			
rs12087868	*GSTM3*	1	109734640	hsa-miR-2115-3p	D	-23.8	-23.8	0	0.1915	No Data	
				hsa-miR-361-5p	D	-29.1	-29.1	0			
				hsa-miR-374a-3p	D	-18.5	-18.5	0			
rs140358309	*GSTM3*	1	109734745	hsa-miR-34a-5p	D	-29.2	-29.2	0	0.4066	6	Mrg1::Hoxa9, Myb, THAP1,
				hsa-miR-34c-5p	D	-30.1	-30.1	0			
				hsa-miR-449a	D	-73.4	-73.4	0			
				hsa-miR-449b-5p	D	-28.3	-28.3	0			
				hsa-miR-548au-3p	D	-26.5	-26.5	0			

				hsa-miR-942-3p	D	-20.6	-20.6	0			
rs184879482	*GSTM3*	1	109734880	hsa-miR-3126-3p	D	-26.3	-26.3	0	0.5104	5	AP-2, Esr2, Pbx3, ZBRK1
				hsa-miR-3176	D	-97.0	-97.0	0			
				hsa-miR-3190-5p	D	-32.4	-32.4	0			
				hsa-miR-3922-3p	D	-31.3	-31.3	0			
rs145451237	*GSTM3*	1	109735074	hsa-miR-302b-5p	D	-27.6	-28.0	-0.4	0.1014	No Data	GCNF,PRDM1,RXRA
				hsa-miR-302d-5p	D	-24.7	-27.0	-2.3			
				hsa-miR-365a-3p	D	-24.7	-24.7	0			
				hsa-miR-365b-3p	D	-24.7	-24.7	0			
				hsa-miR-6886-3p	D	-33.7	-33.7	0			
				hsa-miR-938	D	-64.8	-64.8	0			
rs55976937	*GSTM3*	1	109735215	hsa-miR-4704-3p	D	-22.3	-22.3	0	0.7308	6	ERalpha-a, Ik-1, Maf, Nrf-2, TCF11::MafG
				hsa-miR-500b-3p	D	-29.2	-29.2	0			
				hsa-miR-6879-3p	D	-29.5	-29.5	0			
rs61799820	*GSTM3*	1	109735258	hsa-miR-1301-3p	D	-36.6	-36.6	0	0.4003	No Data	GR, Irf, NF-E2, Zbtb3
				hsa-miR-3671	D	-51.4	-51.4	0			
				hsa-miR-4660	D	-66.0	-66.0	0			
				hsa-miR-5047	D	-80.8	-80.8	0			
				hsa-miR-607	D	-82.6	-85.2	-2.6			
rs114591021	*GSTM3*	1	109735430	hsa-miR-146a-3p	D	-23.7	-23.7	0	0.8932	6	
				hsa-miR-6819-3p	D	-28.9	-28.9	0			
				hsa-miR-6877-3p	D	-32.8	-32.8	0			
rs12058634	*GSTM3*	1	109735432	hsa-miR-146a-3p	D	-23.7	-23.7	0	0.2626	No Data	TCF4
				hsa-miR-6819-3p	D	-28.9	-28.9	0			
				hsa-miR-6877-3p	D	-32.8	-32.8	0			
rs181170041	*GSTM3*	1	109735599	hsa-miR-128-3p	D	-28.8	-28.1	0.7	0.9830	No Data	DMRT4

				hsa-miR-216a-3p	D	-28.0	-28.0	0			
				hsa-miR-3064-3p	D	-25.0	-25.0	0			
				hsa-miR-3681-3p	D	-25.5	-25.5	0			
rs56308184	*GSTM3*	1	109735699	hsa-miR-5693	D	-59.6	-59.6	0	0.8276	6	ATF3, CHOP::CEBPalpha, Maf, SREBP, Zbtb3
				hsa-miR-6499-3p	D	-26.8	-26.8	0			
rs112423011	*GSTM3*	1	109735897	hsa-miR-150-5p	D	-26.1	-26.1	0	0.9512	6	BCL, CTCF, EBF, Ets, Ik-1, PLAG1, SETDB1, ZBTB7A,Zfp740
				hsa-miR-186-3p	D	-24.5	-24.5	0			
				hsa-miR-6778-3p	D	-25.1	-25.1	0			
rs191657187	*GSTM3*	1	109736113	hsa-miR-5583-3p	D	-21.1	-21.1	0	0.4342	6	EWSR, Elf3, Elf5, Maf, NERF1a, PU.1, STAT, p300
				hsa-miR-651-3p	D	-20.3	-20.3	0			
rs183587317	*GSTM3*	1	109736135	hsa-miR-2054	D	-25.4	-25.4	0	0.8393	6	CEBPB,Dbx1, En-1, Hlx1, Hoxb6, Nkx6-1, p300
				hsa-miR-3161	D	-64.7	-64.7	0			
rs148199564	*GSTM3*	1	109736187	hsa-miR-138-2-3p	D	-25.4	-25.4	0	0.4112	No Data	Pou2f2, STAT, TATA, YY1
				hsa-miR-4482-3p	D	-26.4	-26.4	0			
				hsa-miR-7159-3p	D	-20.6	-20.6	0			
rs191033711	*GSTM3*	1	109736514	hsa-miR-421	D	-52.4	-52.4	0	0.4489	No Data	CEBPB,p300
				hsa-miR-4709-5p	D	-23.5	-23.5	0			
rs1803688	*GSTM3*	1	109737041	hsa-miR-494-3p	D	-20.0	-20.0	0	0.9306	4	FAC1, Foxd3, Foxo, Pax-4, Sox, Zfp105
rs201374956	*GSTM3*	1	109737061	hsa-miR-346	D	-103.9	-103.9	0	0.4693	4	PU.1,Rad21,SMC3
				hsa-miR-5001-3p	D	-29.9	-29.9	0			
				hsa-miR-6778-3p	D	-25.1	-25.1	0			
				hsa-miR-6791-3p	D	-32.4	-32.4	0			
				hsa-miR-6829-3p	D	-30.9	-30.9	0			

				hsa-miR-6836-3p	D	-29.4	-29.4	0			
rs1537236	*GSTM3*	1	109736350	miR-182	D	-94.5	-94.5	0	0.5813	6	Sox
				miR-96	D	-67.3	-67.3	0			
rs3814309	*GSTM3*	1	109734781	miR-346	D	-103.9	-103.9	0	0.3514	6	
				miR-4325	D	-25.8	-25.8	0			
				miR-1271	D	-81.7	-81.7	0			
				miR-3130-3p	D	-29.7	-29.7	0			
rs1109138	*GSTM3*	1	109735327	miR-302f	D	-38.6	-38.6	0	0.9777	6	HEN1
				miR-191*	D	-85.6	-85.6	0			
rs2234696	*GSTM3*	1	109736980	miR-3143	D	-51.6	-51.6	0	0.9638	4	
rs11807	*GSTM5*	1	109718120	miR-451	D	-59.7	-59.7	0	0.9855	1d	Pax-5,VDR
rs183054770	*GSTM5*	1	109717447	hsa-miR-2116-3p	D	-30.0	-30.0	0	0.3011	5	EWSR1-FLI1, MAZ, NRSF, PU.1, Rad21, SMC3
rs150528860	*GSTM5*	1	109717454	hsa-miR-1470	D	-76.6	-76.6	0	0.959	5	Maf, Myc, Rad21, SP1, TFII-I, ZNF263
				hsa-miR-4268	D	-66.1	-66.1	0			
				hsa-miR-4667-3p	D	-32.5	-32.5	0			
rs112412754	*GSTM5*	1	109717634	hsa-miR-3134	D	-53.0	-53.0	0	0.9343	2b	GR,Pbx-1
rs116803889	*GSTM5*	1	109717654	hsa-miR-7-5p	D	-22.9	-22.9	0	0.9427	2b	STAT
rs146165082	*GSTM5*	1	10971803	hsa-miR-3915	D	-63.9	-63.9	0	0.7422	4	GATA, GR, Irf, Nanog
				hsa-miR-3928-3p	D	-30.7	-30.7	0			
rs191030500	*GSTM5*	1	109718041	hsa-miR-128-3p	D	-26.6	-26.6	0	0.1881	3a	
				hsa-miR-216a-3p	D	-30.9	-30.9	0			
				hsa-miR-3681-3p	D	-29.0	-29.0	0			
rs41283502	*GSTM5*	1	109718067	hsa-miR-3663-3p	D	-33.3	-33.3	0	0.5230	4	NF-I,NRSF,Sin3Ak-20
				hsa-miR-6501-3p	D	-30.2	-30.2	0			
rs56068346	*GSTM5*	1	109718196	hsa-miR-4436a	D	-88.3	-88.3	0	0.3740	5	GR,Mrg,T3R
				hsa-miR-5000-3p	D	-23.4	-23.4	0			

rs140179217	*GSTM5*	1	109718208	hsa-miR-211-3p	D	-35.0	-35.0	0	0.5331	5	Pax-1,STAT
				hsa-miR-4270	D	-76.7	-76.7	0			
				hsa-miR-4441	D	-85.3	-85.3	0			
				hsa-miR-4772-5p	D	-22.0	-22.0	0			
				hsa-miR-6754-5p	D	-33.8	-33.8	0			
rs182135541	*GSTM5*	1	109718227	hsa-miR-433-3p	D	-29.1	-29.1	0	0.9899	No Data	E4F1
rs5031031	*GSTP1*	11	67586633	hsa-miR-133a-3p	D	-26.9	-26.9	0	0.6789	3a	Cdx2, Hoxa9, Hoxb, Hoxd, Pbx-1, SP2
				hsa-miR-133b	D	-58.1	-61.7	-3.6			
rs201925035	*GSTP1*	11	67586638	hsa-miR-5680	D	-46.9	-46.9	0	0.4363	3a	Cdx2, Hoxa9, Hoxb, Hoxd, SP2
				hsa-miR-590-3p	D	-12.1	-12.1	0			
rs189192686	*GSTZ1*	14	77331286	hsa-miR-2116-3p	D	-26.0	-26.0	0	0.7901	4	Irf,Pax7
				hsa-miR-4279	D	-64.5	-64.5	0			
rs193058583	*GSTZ1*	14	77331392	hsa-miR-7106-3p	D	-38.1	-31.1	7	0.4524	4	CHD2, GR, NRSF, Pou2f2, ZNF263
rs184715697	*GSTZ1*	14	77331429	hsa-miR-1913	D	-77.7	-77.7	0	0.7669	4	Mrg,TFIIA
				hsa-miR-324-3p	D	-28.2	-28.2	0			
rs145440277	*GSTZ1*	14	77331484	hsa-miR-4792	D	-76.9	-76.9	0	0.7517	5	
rs3742739	*GSTZ1*	14	77331532	hsa-miR-3065-5p	D	-16.8	-16.8	0	0.3771	5	FAC1,Pax-2,RREB-1
				hsa-miR-3529-3p	D	-26.6	-22.9	3.7			
				hsa-miR-5003-5p	D	-22.4	-22.0	0.4			
rs140032706	*GSTM4*	1	109661281	hsa-miR-4268	D	-62.1	-62.1	0	0.1116	5	BDP1, CTCF, NF-E2, PU.1, Rad21, TFII-I, UF1H3BETA
				hsa-miR-483-3p	D	-31.8	-29.8	2			
rs143570685	*GSTM4*	1	109661289	hsa-miR-4437	D	-62.3	-62.3	0	0.9405	5	
				hsa-miR-4674	D	-95.3	-95.3	0			
rs1063578	*GSTM4*	1	109661386	hsa-miR-6881-5p	D	-30.5	-30.5	0	0.9976	5	
rs1063579	*GSTM4*	1	109661397	hsa-miR-142-5p	D	-21.7	-21.7	0	0.983	5	Hoxb13
				hsa-miR-5590-3p	D	-21.1	-21.1	0			

				hsa-miR-6507-3p	D	-25.6	-25.6	0			
				hsa-miR-95-5p	D	-17.6	-17.6	0			
rs3211208	*GSTM4*	1	109661414	hsa-miR-4271	D	-65.3	-65.3	0	0.9997	5	BCL, CTCFL, Irf, MZF1, PU, Pax-5, RXRA, STAT,Sp4, VDR_2
				hsa-miR-4725-3p	D	-32.7	-32.7	0			
				hsa-miR-6780b-5p	D	-35.7	-35.7	0			
				hsa-miR-6783-5p	D	-28.5	-28.5	0			
				hsa-miR-6824-5p	D	-34.9	-34.9	0			
rs187989710	*GSTM4*	1	109661423	hsa-miR-193b-5p	D	-28.3	-28.3	0	0.9903	5	STAT,SZF1-1
				hsa-miR-629-5p	D	-25.2	-25.2	0			
rs3211209	*GSTM4*	1	109661465	hsa-miR-1296-3p	D	-31.0	-31.0	0	0.9202	5	Ets,Ik-1,MZF1::1-4
				hsa-miR-4727-3p	D	-27.4	-27.8	-0.4			
				hsa-miR-1245a	D	-53.6	-53.6	0			
rs192415364	*GSTM4*	1	109661490	hsa-miR-6073	D	-71.4	-71.4	0	0.9914	5	
				hsa-miR-6871-5p	D	-32.6	-32.6	0			
				hsa-miR-8062	D	-54.4	-54.4	0			
				hsa-miR-8079	D	-79.6	-79.6	0			
rs187180038	*GSTM4*	1	109661569	hsa-miR-3184-5p	D	-32.9	-32.9	0	0.3594	5	ERalpha-a, Ets, HNF4, LF-A1, RXRA
				hsa-miR-423-5p	D	-31.6	-31.6	0			
rs1051134	*GSTM4*	1	109661656	hsa-miR-6508-3p	D	-27.6	-27.6	0	0.4909	5	CTCFL, E2A, INSM1, RXRA
				hsa-miR-6762-5p	D	-42.3	-37.5	4.8			
				hsa-miR-6845-5p	D	-33.0	-31.2	1.8			
rs1063585	*GSTM4*	1	109661657	hsa-miR-1909-3p	D	-36.6	-36.6	0	0.9266	5	RXRA
				hsa-miR-6508-3p	D	-27.6	-27.6	0			
				hsa-miR-6722-3p	D	-42.3	-42.3	0			
				hsa-miR-6762-5p	D	-42.3	-39.6	2.7			
				hsa-miR-6845-5p	D	-33.0	-31.2	1.8			

rs3204618	*GSTM4*	1	109661681	hsa-miR-383-5p	D	-25.4	-25.4	0	0.9612	5	
				hsa-miR-4772-5p	D	-24.3	-24.3	0			

1. Chromosome
2. Function Class
3. (kcal/mol)
